# Neglected Tropical Diseases: Epidemiology and Global Burden

**DOI:** 10.3390/tropicalmed2030036

**Published:** 2017-08-05

**Authors:** Amal K. Mitra, Anthony R. Mawson

**Affiliations:** Department of Epidemiology and Biostatistics, School of Public Health, Jackson State University, Jackson, PO Box 17038, MS 39213, USA; anthony.r.mawson@jsums.edu

**Keywords:** epidemiology, risk factors, global burden, DALYs, NTDs

## Abstract

More than a billion people—one-sixth of the world’s population, mostly in developing countries—are infected with one or more of the neglected tropical diseases (NTDs). Several national and international programs (e.g., the World Health Organization’s Global NTD Programs, the Centers for Disease Control and Prevention’s Global NTD Program, the United States Global Health Initiative, the United States Agency for International Development’s NTD Program, and others) are focusing on NTDs, and fighting to control or eliminate them. This review identifies the risk factors of major NTDs, and describes the global burden of the diseases in terms of disability-adjusted life years (DALYs).

## 1. Introduction

Neglected tropical diseases (NTDs) are a group of bacterial, parasitic, viral, and fungal infections that are prevalent in many of the tropical and sub-tropical developing countries where poverty is rampant. According to a World Bank study, 51% of the population of sub-Saharan Africa, a major focus for NTDs, lives on less than US$1.25 per day, and 73% of the population lives on less than US$2 per day [1]. In the 2010 Global Burden of Disease Study, NTDs accounted for 26.06 million disability-adjusted life years (DALYs) (95% confidence interval: 20.30, 35.12) [2]. In addition to their impact on health, NTDs contribute to an immense social and economic burden resulting from social stigma, physical disabilities, disfigurement, blindness, discrimination, loss of social status, malnutrition, growth failure, and impaired cognitive development. All of these interrelated outcomes perpetuate the cycle of poverty by preventing individuals from leading productive lives, and by adversely affecting families, communities, and countries as a whole. However, many of these diseases are preventable, and could be eliminated with improved sanitation, vector control, available treatments, and mass drug administration (MDA) campaigns.

For the programmatic point of view, the World Health Organization (WHO) classified NTDs into two groups: preventive chemotherapy and transmission control (PCT) NTDs, and innovative and intensified disease management (IDM) NTDs [3]. The most prominent examples of NTDs that have been allocated to the PCT group are lymphatic filariasis, onchocerciasis, schistosomiasis, and soil-transmitted helminthiasis; the main tool for their control is the periodic administration of efficacious, safe, and inexpensive (usually donated) drugs to entire at-risk populations. IDM, on the other hand, focuses on those NTDs that currently lack appropriate tools for large-scale use. These diseases include Buruli ulcer, Chagas disease, human African trypanosomiasis, and leishmaniasis [4].

On 30 January 2012, a number of organizations including the WHO, Bill & Belinda Gates Foundation, United States Agency for International Development (USAID), World Bank, United Kingdom Department for International Development (DFID), pharmaceutical companies, and government officials from donor and endemic countries met together at a meeting entitled “Uniting to Combat NTDs: Ending the Neglect and Reaching the 2020 Goals”, and set targeted goals for NTDs [5].

Climate change and global warming are increasing the likelihood and spread of many vector-borne diseases, including malaria, dengue fever, Chagas disease, leishmaniasis, filariasis, onchocerciasis, schistosomiasis, and trypanosomiasis [6]. At the 70th World Health Assembly (WHA) held in Geneva, Switzerland in May 2017, a resolution was adopted on a Global Vector Control Response for 2017–2030 that aims to prevent, detect, report and respond to outbreaks of vector-borne diseases worldwide through an integrated, comprehensive approach [7].

On 23 May 2017, the World Intellectual Property Organization (WIPO) Re:Search launched a new five-year roadmap to guide public–private consortium activities, including research, capacity-building, and outreach efforts in the fight against NTDs, malaria and tuberculosis, which together cause devastation and disproportionately affect the poorest and most disadvantaged people [8]. The Carter Center is also taking initiatives to eradicate or eliminate several NTDs, including guinea worm disease in South Sudan, Mali, Chad, and Ethiopia, and lymphatic filariasis in Nigeria, Ethiopia and Hispaniola [9].

This review summarizes current data and information on the epidemiology, risk factors, and global burden of major NTDs, and suggests future research and public health measures.

## 2. Epidemiology and Risk Factors

The 2020 roadmap of WHO, joined by others, focuses on 20 NTDs. These diseases include Buruli ulcer, Chagas disease, cysticercosis/taeniasis, dengue fever, dracunculiasis (guinea worm disease), echinococcosis, food-borne trematodiasis, human African trypanosomiasis (HAT) (sleeping sickness), leishmaniasis, leprosy, lymphatic filariasis, onchocerciasis (river blindness), rabies, schistosomiasis, soil-transmitted helminthiasis (ascariasis, hookworm, trichuriasis), trachoma, and yaws (Table 1).

These diseases are also included in CDC action plans [10]. In 2017, at the 10th meeting of the Strategic and Technical Advisory Group for NTDs, the WHO added mycetoma, scabies and snakebite to the NTD list [3]. The London Declaration on NTDs, launched in 2012, proposed to sustain, expand, and extend programs to help eradicate guinea worm disease; eliminate lymphatic filariasis, leprosy, sleeping sickness, and trachoma; and control schistosomiasis, soil-transmitted helminthiasis, Chagas disease, visceral leishmaniasis, and river blindness by the year 2020 [11]. A brief description of the epidemiology and the risk factors of the common NTDs follows.

### 2.1. Buruli Ulcer

Buruli ulcer (BU) is a necrotizing skin disease caused by *Mycobacterium ulcerans*, which is a slow-growing mycobacterium that infects the skin and subcutaneous tissues, giving rise to indolent ulcers. The disease has been reported from more than 30 countries, but especially in West Africa, with 80% of cases from the Ivory Coast, Ghana, Benin and Cameroon [12,13]. In a household survey between February and May 2013 along the Offin River in Ghana, 477 cases with healed BU and eight active cases were detected among 20,390 inhabitants, with an overall prevalence of 2.3% [12]. Approximately 48,373 cases were reported from 20 countries, mostly in West and Central Africa. The risk is highest in children aged 4–14 years, and in people aged over 50 years [13].

Transmission of the disease has been linked with contaminated water [14]. Aquatic insects, adult mosquitoes and biting arthropods have been considered possible reservoir species and/or vectors. Regular contact with open surface water (i.e., river, pond, creek, and dam) was associated with higher odds of contracting BU (OR = 9.3, 95% CI: 4.3, 20.0). The most risky daily activities directly or indirectly related to water contact were farming (rice and vegetables) and fishing (OR = 5.6, 95% CI: 2.6, 12.3), contacting water for household supply at surface water points (OR = 3.3, 95% CI: 1.6, 6.6), and washing and/or bathing at surface water points (OR = 2.5, 95% CI: 1.1, 5.6) [13].

WHO recommends daily administration of streptomycin and rifampicin for eight weeks as the treatment of choice. For pregnant women, the combination of rifampicin and clarithromycin is considered the safer option because of contraindication to streptomycin. The only effective control tool is early case detection and treatment to reduce morbidity and associated disabilities that occur as a result of late treatment. In a case study in the Obom sub-district in Ghana, community involvement and social interventions were found to enhance early diagnosis and treatment [15].

### 2.2. Chikungunya

The chikungunya virus (CHIKV), spread by *Aedes (Ae.) aegypti* mosquitoes to humans, is now classified as a category C priority pathogen by the US National Institute of Allergy and Infectious Diseases, as it has spread to over 40 countries worldwide [16]. Another vector, the *Ae. albopictus* mosquito, thrives in a wider range of water-filled breeding sites than the aegypti mosquito, which can include coconut husks, cocoa pods, bamboo stumps, tree holes and rock pools in addition to artificial containers and vehicle tires. In recent decades, *Ae. albopictus* has spread from Asia and become established in areas of Africa, Europe and the Americas. The continued transmission of chikungunya in Colombia and other Latin American countries raises a public health concern [17]. Vertical transmission between mother and fetus, however, has been observed in some cases [18]. Confirmed cases have been reported from 24 countries in Africa, 20 in Asia, 44 in America, and 10 countries in Oceania/Pacific Islands. The most recent outbreaks were documented in Réunion, Mauritius, India, coastal Italy, and Bangladesh. In India, outbreaks of chikungunya were recorded from 13 of the 30 districts of Orissa. Attack rates ranged from 0.4% to 50.76% in the different villages [16].

There is a positive linear relationship between the incidence of CHIKV and mean temperature ranging from 10–25 °C [19]. In face-to-face interviews with more than 500 individuals with 314 (62%) seropositive cases in southern Thailand, the risk factors associated with CHIKV infection were outdoor activities daily for at least eight hours, and having a nearby garbage pile. The protective factors for symptomatic infection were age ≥58 years, and having a high level of formal education [20].

Treatment usually is for the symptoms, and includes taking sufficient rest, and taking more fluid and food, and medicines to relieve pain (paracetamol for example). Aspirin should be avoided. Prevention of chikungunya virus infection is by avoiding mosquito bites: air conditioning or window/door screens to keep mosquitoes outside; sleeping inside a mosquito net, wearing long-sleeved shirts and long pants when weather permits, and using repellents containing DEET, picaridin, IR3535, and oil of lemon eucalyptus or para-menthane-diol (or menthoglycol) [21].

### 2.3. Chagas Disease

The causative agent of Chagas disease is a parasite, *Trypanosoma cruzi*, transmitted by *Triatoma infestans* (or kissing bug), the primary vector of the disease [22]. The disease is found in North America, Central America, and South America. The most important consequence of *T. cruzi* infection is cardiomyopathy, which occurs in 20–30% of infected persons. The vectors live in the cracks in mud walls and thatched roofs of rustic rural houses, causing repeated infections. The estimated global prevalence of *T. cruzi* infection declined from 18 million in 1991, when the first regional control initiative began, to 5.7 million in 2010. Most cases in the United States are imported from Latin America, with an estimated 300,000 infected residents living in the U.S. Outbreaks of orally transmitted *T. cruzi* infection through food or drink contaminated with vector feces appear to be associated with a higher incidence of myocarditis and a higher case-fatality rate than vector-borne infections [23].

Three studies that tested anti-*T. cruzi* seropositivity found strong and statistically significant associations between socioeconomic status with infection, with two to three times higher odds of infection among people of lower than higher socioeconomic strata [24]. A study of pregnant women in Colombia showed about a 20-fold increase in the odds of contracting the infection among women without completed primary education, compared with university-educated women (OR = 19.6; 95% CI, 2.5–152.2) [24].

All patients with acute Chagas disease, including infants with congenital infection and persons with reactivation of chronic infections due to immunosuppression, should be treated with either benznidazole or nifurtimox. However, efficacy of medicines decreases as the duration of the infection lengthens [25]. More research is needed for prophylactic treatment of chronic infection in immunosuppressed persons.

### 2.4. Dengue Fever

Dengue fever (DF) is caused by four serotypes of a virus (DENV), which is transmitted by mosquitoes, primarily *Aedes aegypti* and *Ae*. *albopictus.* Approximately 390 million people are exposed to DENV each year, resulting in 96 million annual cases of viral-associated disease globally, while approximately 3.6 billion people living in the tropical and sub-tropical regions are at risk of infection [26,27]. According to the WHO, approximately 500,000 people develop severe disease each year, and among them, about 1250 (2.5%) die [27].

The first identified epidemic of DF and dengue hemorrhagic fever (DHF) in Bangladesh took place during the monsoon season of 2000, and resulted in 5521 officially reported cases, with 93 fatalities [28]. Risk of the positive seroprevalence of DENV was significantly associated with increasing age (OR = 4.1 for the age group 12–44 years; OR = 5.9 for age group 45 years and older compared with younger age group <12 years, *p* <0.001). There was a protective effect on seroprevalence among those who did not have indoor potted plants compared with those who did (OR = 0.53, *p* = 0.004) [28]. Holding water in containers of potted plants is a good source of breeding of *A. aegypti* mosquitoes. In another study in Machala, Ecuador, older age, a female head of the household, and poor household conditions were significantly associated with the presence of dengue fever [29]. People living in an unhygienic house, or in a house discharging sewage directly to the ponds were 3·4 times and 4·3 times, respectively, more likely to be associated with DF/DHF [30].

There is no effective treatment for dengue fever. Supportive care with analgesics, fluid replacement, and bed rest is usually sufficient. Acetaminophen may be used to treat fever and relieve other symptoms. Aspirin, nonsteroidal anti-inflammatory drugs (NSAIDs), and corticosteroids should be avoided. Management of severe dengue fever requires careful attention to fluid management and proactive treatment of hemorrhage, such as platelet transfusion or whole blood transfusion [27,28].

### 2.5. Dracunculiosis (or Guinea Worm Disease)

Dracunculiasis, commonly known as guinea worm disease, is caused by a two- to three-foot long worm, *Dracunculus medinensis*. People contract the disease by drinking contaminated water from open sources, such as stagnant ponds that contain immature parasites in tiny copepods (water fleas) [31]. During the 1970s, the disease was prevalent in the rural areas of India, the Islamic Republic of Iran, Pakistan, Saudi Arabia, Yemen, and East and West Africa. The Global Guinea Worm Eradication (Dracunculiasis) Program has made spectacular progress since it began in the 1980s. From an estimated 3.5 million cases in 1986, it has dropped to only 25 cases in 2016 [32,33]. As of 2013, 197 countries have been certified free from dracunculiasis [34]. At the end of 2016, 17 of the 21 previously endemic countries had stopped transmission of the disease, out of which 15 have been certified free of transmission by the WHO [32]. Currently, four endemic countries remain: Chad, Ethiopia, Mali, and South Sudan, which reported a total of 25 human cases in 2016 [32,33]. Mali did not report any human infection in 2016. Kenya and Sudan, which were previously endemic, have not reported a case for at least three years. The use of open stagnant water sources such as man-made ponds and sometimes shallow or step wells are the main sources of transmission [35]. In the Sahelian zone, transmission generally occurs in the rainy season (May to August). In the humid savanna and forest zone, the peak occurs in the dry season (September to January) [35].

No specific drug is used to treat dracunculiasis. The mainstay of treatment is the extraction of the adult worm from the patient using a stick at the skin surface and wrapping or winding the worm a few centimeters per day. The wound is cleaned, and gentle traction is applied to the worm to slowly pull it out. Because the worm is long, full extraction can take several days to weeks. Metronidazole or thiabendazole (in adults) is usually adjunctive to stick therapy. The prevention of the disease can be achieved by promoting health education and behavioral change of the people, such as drinking safe water and avoiding wading into water [25].

In 1981, WHO’s decision-making body, the World Health Assembly, adopted a resolution (WHA 34.25) recognizing that the International Drinking Water Supply and Sanitation Decade presented an opportunity to eliminate dracunculiasis. In 1986, the Carter Center joined the battle against the disease, in partnership with WHO and UNICEF. For the guinea worm eradication program, the Carter Center received a $40 million grant from the Gates Foundation. The UK Department for International Development (DFID) pledged a £10 million (approximately US$15 million) to the Carter Center to support the guinea worm eradication campaign, and its support will be matched by the Gates Foundation [9].

### 2.6. Human African Trypanosomiasis (or Sleeping Sickness)

Human African trypanosomiasis (HAT), also known as sleeping sickness, is a vector-borne parasitic disease caused by infection with one of two parasites: *Trypanosoma brucei gambiense* and *T. b. rhodesiense*, and transmitted by insect vectors, tsetse flies [36,37]. An estimated 60 million people are at risk of both forms of parasites in sub-Saharan Africa [36]. According to Malvy and Chappuis [38], in 2010, *Trypanosoma brucei gambiense* was focally endemic in 24 countries of Western and Central Africa, mainly Angola, the Democratic Republic of Congo (DRC), the Central African Republic, Chad, Ivory Coast, Guinea, southern Sudan, and northwest Uganda. In 2013, out of a total of 6228 reported new gambiense HAT cases, 5647 (more than 90%) were clustered in the DRC [39].

Pentamidine is the recommended drug for first stage *T. b. gambiense* infection. The other drugs used to treat African trypanosomiasis include suramin, melarsoprol, eflornithine, and nifurtimox. There is no vaccine or drug for prophylaxis against HAT. Preventive measures are aimed at minimizing contact with tsetse flies. Control of HAT rests on two strategies: reducing the disease reservoir, and controlling the tsetse fly vector. Reducing the reservoir of infection is more difficult for *T. b. rhodesiense*, since there are a variety of animal hosts. Vector control is the primary strategy in use. This is usually done with traps or screens, in combination with insecticides and odors that attract the flies [40].

Coordinated efforts have been made by the National Sleeping Sickness Control Program and WHO in data collection, reporting, management, and mapping of HAT distribution and risk in the DRC. Less than one third of the land area in the DRC (i.e., 715 thousand km^2^) and approximately half of the population (i.e., 36.6 million) are currently estimated to be at various levels of HAT risk [41]. Rapid detection and effective treatment of newly detected HAT patients is one of the cornerstones of HAT control.

### 2.7. Leishmaniasis

Visceral leishmaniasis (VL) or kala-azar is a vector-borne parasitic disease caused by *Leishmania donovani*, which is transmitted from man to man by the sand fly *Phlebotomus argentipes* [42]. Of the 200,000 to 400,000 new cases of VL worldwide, more than 90% occur in six countries: India, Bangladesh, Sudan, South Sudan, Ethiopia and Brazil [41,42,43]. The risk of seroconversion and disease was significantly increased in individuals aged 14–24 years old [42]. Higher socioeconomic status was associated with a decreased risk of seroconversion. In the American region, the estimated annual incidence of VL is 4500 to 6800 cases; of these, 4200 to 6500 cases (>95%) occurred in Brazil alone [44].

Treatment of leishmaniasis depends on the type of the disease. The skin sores of cutaneous leishmaniasis usually heal on their own, even without treatment. The best way to prevent mucosal leishmaniasis is to ensure adequate treatment of the cutaneous infection. Severe (advanced) cases of visceral leishmaniasis typically are fatal [45]. Possible treatments for cases include oral ketoconazole (Nizoral, Extina, Xolegel, Kuric), intravenous pentamidine, or liposomal amphotericin B.

Three neighboring countries in Southeast Asia—Bangladesh, India, and Nepal—joined the WHO’s kala-azar elimination program in 2005. All three countries have made significant progress towards the targets. The number of cases has decreased by 59%, mortality by 85% and case fatality by 61%. Nepal has eliminated the disease at district level, and maintained the situation for the past two years. Bangladesh has achieved the elimination target in 90% of endemic sub-districts. India has achieved the target in more than two-thirds of endemic areas [46].

### 2.8. Leprosy

Leprosy is known as a disease of poverty. Only in the poorest areas of the world is leprosy, an infectious disease caused by *Mycobacterium leprae,* still endemic. Brazil, India, Nepal, Myanmar, Madagascar, and Mozambique are responsible for almost 90% of the leprosy cases registered worldwide [47]. The worldwide use of multidrugs started in the 1980s, and their free access since 1995 contributed to the drastic decline in the number of new cases [48]. In the poverty-stricken northwestern part of Bangladesh, where The Leprosy Mission Bangladesh operates a leprosy control program, the incidence was still 1.25 per 10,000 inhabitants in 2008 [49]. In a case-control study, a recent period of food shortage and not poverty per se was identified as the only socioeconomic factor significantly associated with clinical manifestations of leprosy disease (OR = 1.79, 95% CI: 1.06, 3.02; *p* = 0.030) [49]. The overall prevalence of clinical leprosy in a study area in Egypt was 24.9/10,000 (95% CI: 16.3, 37.6). Individuals over age 40 were four-fold more likely to develop leprosy (OR = 4, *p* = 0.01) [50]. The risk for leprosy was associated with HLA-DR2 and HLA-DQ1 markers, and these markers appear to increase personal susceptibility to leprosy in this area.

Eighty percent of all leprosy cases in the Americas occur in Brazil. In a case-control study in northeast Brazil, a low education level, having ever experienced food shortages, bathing weekly in open bodies of water (creeks, rivers and/or lakes), and a low frequency of changing bed linen or hammock were all significantly associated with leprosy. Having a BCG vaccination scar was found to be a highly significant protective factor [47].

Several drugs are used in combination in multidrug therapy (MDT). Rifampicin is now combined with dapsone to treat paucibacillary leprosy. Rifampicin and clofazimine are now combined with dapsone to treat multibacillary leprosy. A single dose of combination therapy has been used to cure single lesion paucibacillary leprosy: rifampicin (600 mg), ofloxacin (400 mg), and minocycline (100 mg). The child with a single lesion takes half the adult dose of the three medications [25].

### 2.9. Lymphatic Filariasis

Lymphatic filariasis (LF) is a chronic, disabling and often disfiguring condition due to lymphatic obstruction, which results in marked swelling of the lower extremities (elephantiasis) and genitals (hydrocele, causing scrotal swelling in men). The disease is caused by parasitic infection from filarial worms. Most of the infections worldwide are caused by *Wuchereria bancrofti*. In Asia, the disease can also be caused by *Brugia malayi* and *Brugia timori*. A wide range of mosquitoes can transmit the parasite, depending on the geographic area. In Africa, the most common vector is *Anopheles* and in the Americas, it is *Culex quinquefasciatus*. In the Pacific and in Asia, *Aedes* and *Mansonia* can transmit the infection [51]. Global estimates suggest that 120 million people are affected in 80 countries throughout the tropics and sub-tropics, with people at risk exceeding 1.3 billion [52]. In sub-Saharan countries alone, 46–51 million people suffer from LF [1].

A case study in Congo demonstrated an increased risk for males (OR = 2.0, 95% CI: 1.3, 3.0) and for people who hunt or fish (OR = 1.5, 95% CI: 1.0, 2.4) and a protective effect of latrines (OR = 0.5, 95% CI: 0.4, 0.8) [53]. Among males, those hunting or fishing at night had an increased risk for antigenemia (OR = 1.9, 95% CI: 1.1–3.5), while the use of latrines was protective (OR = 0.5, 95% CI: 0.3–0.9). For females, bed nets were protective (OR = 0.4, 95% CI: 0.1–0.9).

Diethylcarbamazine (DEC) is the drug of choice. However, DEC should not be administered to patients who may also have onchocerciasis, as DEC can worsen onchocercal eye disease. Ivermectin kills only the microfilariae, but not the adult worm. Some studies have shown adult worms can be killed with doxycycline treatment (200 mg/day for four to six weeks) [25].

### 2.10. Onchocerciasis (or River Blindness)

Onchocerciasis (river blindness) is a parasitic disease caused by the nematode *Onchocerca volvulus*, transmitted by blackflies. The disease causes severe itching, skin lesions, and vision impairment, including blindness. Onchocerciasis is endemic in parts of Africa, Latin America, and Yemen, but over 99% of all current cases are found in sub-Saharan Africa [54,55]. Onchocerciasis (river blindness) is one of the NTDs targeted for elimination. Studies in Mali and Senegal proved the feasibility of elimination with ivermectin (Stromectol) administration [55]. However, elimination of the diseases remains a challenge in some “hot spots” where the treatment with ivermectin increases the risk of serious adverse events in individuals with high parasitemia [56]. Non-compliance with MDA, limited access to annual preventive chemotherapy, and inability of health care providers to adequately diagnose the disease are among the challenges of containing and eradicating the disease in the Republic of Cameroon [57].

### 2.11. Rabies

Rabies is fatal, and one of the most important reemerging zoonotic diseases throughout the world. Transmission of the virus usually occurs by the bite of rabid animals. South Asian countries contribute to more than half of the global burden of rabies. India is a major contributor to the global rabies burden, being responsible for 17,000–20,000 of the 55,000–70,000 deaths that occur globally each year [58,59]. In Nepal, reported human deaths due to rabies are about 10–100 per year [60]. Pre-exposure prophylaxis is recommended for individuals who are at increased risk of exposure to the rabies virus, such as animal handlers, laboratory technicians, and veterinarians in endemic countries. The WHO-recommended post-exposure prophylaxis consists of immediate and proper wound management and a course of antirabies vaccine, and for high-risk exposures, administration of rabies immunoglobulins [60].

### 2.12. Scabies

Scabies is a common parasitic infection caused by the mite *Sarcoptes scabiei*. The worldwide prevalence has been estimated at about 300 million cases yearly. Unhygienic living conditions and poor personal hygiene are favorable for scabies. Mass treatment with an antiparasitic cream and personal hygiene can effectively reduce the prevalence. In a controlled study among young students in madrasahs (religious schools) in Dhaka, Bangladesh, the prevalence of scabies was over 60% [61]. After intervention with mass treatment of all students, teachers and staff of the study areas with topical application of 5% permethrin cream, weekly health education classes, and daily monitoring of students for five key personal hygiene practices, the prevalence rate of scabies in the intervention areas dropped to 5%, compared to 50% in the control areas, over a four-month period [61].

MDA programs have been attempted to use ivermectin to control scabies in endemic communities around the world [62]. However, the superiority of such programs over alternative topical treatment is questionable because (1) pregnant women must be screened out; (2) giving ivermectin on an empty stomach (for better absorption) is a challenge in community-based programs, (3) the drug is recommended for people who have crusted scabies, or for people who do not respond to the prescription lotions and creams, and (4) its use is not recommended in those under five years of age, yet this is the most vulnerable group [62].

### 2.13. Schistosomiasis

Schistosomiasis, or bilharzia, is a parasitic infection caused by any of several trematode worms (flukes) of the genus *Schistosoma*. Snails are the intermediate hosts. There are five species that are known to infect humans: S. *mansoni*, S. *intercalatum*, S. *haematobium*, S. *japonicum*, and S. *mekongi*. In Africa, S. *mansoni* and S. *haematobium* are predominant throughout the continent, while S. *intercalatum* is found in certain areas of central and western Africa. S. *mansoni* is also found in Latin America and the Caribbean. S. *japonicum* and S. *mekongi* are mostly confined to Asia and the Pacific [59]. Globally, at least 230 million people are estimated to have schistosomiasis, the majority of which are from sub-Saharan Africa [63,64,65]. Factors responsible for persistent transmission of the disease in sub-Saharan countries include climate changes and global warming, proximity to water bodies, irrigation and dam construction, occupational activities such as fishing and farming, and poverty [65].

The recommended strategy for schistosomiasis is mass treatment with praziquantel, which effectively clears the body of worms, but reinfection is common due to the nature of the parasites’ transmission and human behavior. Despite considerable efforts made to control schistosomiasis using integrated approaches, including repeated mass chemotherapy using praziquantel, public health education focusing on behavior changes towards risk factors, improved sanitation/hygiene, and treatment of snail habitats, the disease remains a serious public health problem in sub-Saharan Africa [65,66].

### 2.14. Soil-Transmitted Helminthiasis

Soil-transmitted helminth (STH) infection is caused by intestinal nematodes, of which the three major parasite types are *Ascaris lumbricoides* (roundworm), *Trichuris trichiura* (whipworm), and two species of hookworm (*Necator americanus* and *Ancylostoma duodenale*). STH is transmitted to humans through fecally-contaminated soil. According to the Global Atlas of Helminth Infection (GAHI), at least 120 countries across the tropics and subtropics are endemic, and at least 1.3 billion people were estimated to be infected with at least one STH species in 2010 [67]. South Asia, Southeast Asia, and sub-Saharan Africa are the regions with the highest prevalence. In a study using a field method of detecting STH eggs in soil in 2015, the prevalence of any STH (*Ascaris*, *Trichuris* or hookworm) egg in soil was 78% in Bangladesh, and 37% in Kenya [68]. Morbidity control through preventive chemotherapy (PC) has been embraced by endemic countries, the WHO, and by partners as a clear and achievable goal [69]. The WHO-recommended medicines—albendazole (400 mg) and mebendazole (500 mg)—are effective, inexpensive and easy to administer by non-medical personnel [26]. Globally, PC has reduced the number of individuals with morbid STH infections by 85%. Data suggests that after 10 years of annual PC interventions, STH-associated morbidity can be virtually eliminated [69].

### 2.15. Trachoma

Trachoma is a bacterial eye infection caused by the bacterium *Chlamydia trachomatis*. It is spread from person to person through contact with infected eye and nose secretions, often through hands and clothing, and is also spread by eye-seeking flies. Repeated infection can develop into a condition known as trichiasis, in which scarring and inward turning of the eyelid causes the eyelashes to scrape against the cornea of the eye. In a typical endemic setting, repeated chlamydial infection of the conjunctiva starts early in life. This can initiate recurrent episodes of chronic conjunctival inflammation, characterized by the formation of lymphoid follicles. These are most easily seen in the upper tarsal conjunctival surface, known as pannus formation [70]. If left untreated, this painful condition can result in permanent blindness [71].

Globally, 1.2 billion people live in endemic areas, 40.6 million people are suffering from active trachoma, and 48.5% of the global burden of active trachoma is concentrated in five countries: Ethiopia, India, Nigeria, Sudan and Guinea. Overall, Africa is the most affected continent, with 27.8 million (68.5% of the 40.6 million) cases of active trachoma [72].

Trachoma is commonly found in areas with limited access to adequate water, sanitation, and basic hygiene (WASH). In a study in southern Sudan, risk factors such as an unclean face, less frequent face washing, cattle ownership, and increasing fly density were found to be independently associated with severity of active trachoma, after adjusting for age and sex [73]. With the help of an MDA program with azithromycin, the target of trachoma elimination set in 1998 appears to be a realistic and achievable goal [71].

## 3. Global Burden

**Figure 1:**
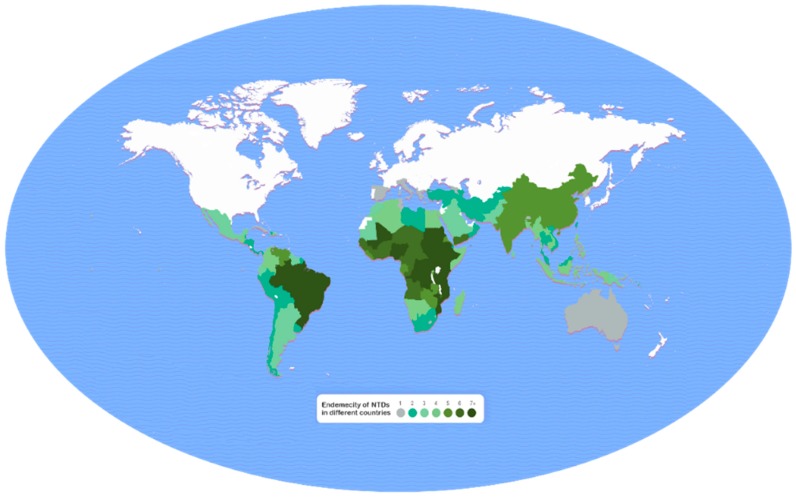
Prevalence of neglected tropical diseases (NTDs) by country. The burden of NTDs in different countries is expressed as number of NTDs prevalent (ranging from one to seven or more). Modified from: United to Combat. Burden map—Neglected Tropical Diseases [74].

Disease burden was expressed in terms of disability-adjusted life years (DALYs), which was calculated using the following formula: DALY = years of life lost (YLL) + years of life lived with disability (YLD). The global distribution of NTDs is shown in Figure 1 [74], in which the vast majority of cases (seven or more NTDs) are located in Brazil and in some countries in Central and East Africa, and in Yemen, followed by cases (five or more) in India, Bangladesh, and China. Based on the data collected by WHO in 14 of the targeted 20 NTDs [75], the total worldwide burden is 25,135.59 thousand years lost, as estimated by DALYs in 2015 (Table 2). This enormous burden of the NTDs is mostly shared by soil-transmitted helminthiasis (STH) (4443.47 thousand years), schistosomiasis (3513.85 thousand years), dengue fever (2610.08 thousand years), lymphatic filariasis (2070.85 thousand years), cysticercosis (a larval tapeworm infection) (1135.57 thousand years), human rabies (1672.17 thousand years), leishmaniasis (1356.46 thousand years), onchocerciasis (1135.57 thousand years), and foodborne trematodiasis (1066.34 thousand years). These data should be interpreted with caution because data for years of life lost (YLL) were not available for trichuriasis, hookworm infection, lymphatic filariasis, onchocerciasis, and trachoma for 2015.

Bhutta et al. (2014) [76] reported a similar picture of the global burden of NTDs using the WHO DALY estimates for 2010 (Table 2). Obviously, the burden of the diseases in numerical terms have changed over time. However, the major diseases contributing to the overall burden remained similar.

In another report from the Global Burden of Disease Study 2010 [2], the estimated DALYs of the NTDs was 26.06 million years, with the highest burden for soil-transmitted helminthiasis, followed by leishmaniasis, schistosomiasis, lymphatic filariasis, and food-borne trematodiasis. However, in an earlier report of NTD burden by Mathers et al. (2007) [77], there were some noticeable differences: lymphatic filariasis accounted for the highest burden of 5777 thousand DALYs, followed by soil-transmitted diseases (3796 thousand years), trachoma (2329 thousand years), leishmaniasis (2090 thousand years), schistosomiasis (1702 thousand years), and trypanosomiasis (1525 thousand years), globally in 2002.

In addition to the above diseases, chikungunya has posed a public health threat in recent years. Recent studies have shown that the DALYs lost during the 2006 epidemic of chikungunya in India totaled 25,588 with an overall burden of 45.26 DALYs per million people [78]. For the 2014 epidemic of Colombia, an estimated total DALYs lost was 40.44 to 45.14 per 100,000 population [17].

## 4. Recommendations

In order to achieve the 2020 roadmap against NTD, further research is needed for their effective control. This includes the need for newer and safer drugs, vector control, personal hygiene, and the development of vaccines. Based on this review, we suggest the following:

1. Community awareness and early diagnosis of Buruli ulcer were found to be effective tools in reducing complications of the disease in rural areas of West and Central Africa [16], but more studies are needed to elucidate the mechanism by which *M. ulcerans* is transmitted from the environment to humans.
2. Integrated vector management through the elimination of breeding sites, use of anti-adult and anti-larval measures and personal protection will help to prevent outbreaks of several vector-borne diseases including chikungunya, dengue fever, and HAT.
3. Because the efficacy of medicines in Chagas disease decreases with increased chronicity of the disease, early intervention and treatment of the acute phase of infection would be efficacious than treatment of the chronic stage of the disease. There is a need for more effective, safer, and easier-to-use medicines for both phases of Chagas disease.
4. Over 200 countries so far have been certified free of dracunculiasis, or guinea worm disease. Health education and behavioral change are effective tools in disease prevention [25].
5. Early detection and treatment of new cases and vector control are essential to the control of HAT [40,41].
6. Treatment with ivermectin remains a challenge in individuals with onchocerciasis, especially those with high rate of parasitemia, because of the side effects and poor compliance of the drug in such cases [56]. Similarly, ivermectin is not recommended universally in the treatment of scabies. Future studies should aim at finding newer and safer medicines.

In terms of DALY, the major burden of NTDs remains due to soil-transmitted diseases, schistosomiasis, dengue fever, and lymphatic filariasis. In fact, dengue fever epidemics have created havoc in many developing countries in recent years. The need for a dengue fever vaccine is evident. Priorities in public health action plans are needed for those diseases which are causing most disabilities, and thereby, the most productive years lost among the other NTDs.

## 5. Conclusions

As the global fight against NTDs continues, more cutting-edge public health policies and research are needed to find effective drugs and vaccines. Partnerships involving major donor agencies, charitable organizations, NGOs, government leaders, pharmaceutical companies, and other key stakeholders are crucial in the fight against NTDs and enabling access to treatment for millions of people worldwide. Several partners have already announced new funding in order to combine efforts to prevent and to find new treatments for NTDs. Although progress is being made against NTDs, continued success depends on a policy environment, considerable political support, and collaboration from all participants.

## Figures and Tables

**Table 1:** List of Neglected Tropical Diseases by Centers for Disease Control and Prevention (CDC) and World Health Organization (WHO).

Disease	CDC	WHO
Buruli ulcer (*Mycobacterium ulcerans* infection)	+	+
Chikungunya ^a^	–	+
Chagas disease	+	+
Cysticercosis	+	+
Dengue fever	+	+
Dracunculiosis (or guinea worm disease) ^b^	+	+
Echinococcosis	+	+
Fascioliasis	+	+
Foodborne trematodiasis ^a^	–	+
Human African trypanosomiasis (or sleeping sickness)	+	+
Leishmaniasis (or kala-azar)	+	+
Leprosy	+	+
Lymphatic filariasis ^b^	+	+
Mycetoma	+	+
Onchocerciasis (or river blindness) ^b^	+	+
Rabies	+	+
Schistosomiasis ^b^	+	+
Soil-transmitted helminthiasis ^b^	+	+
Trachoma ^b^	+	+
Yaws	+	+

Data source: CDC, 2017 [10]; WHO, 2017 [3]; ^a^ Not mentioned under CDC list of neglected tropical diseases (NTDs); ^b^ Diseases that can be controlled or eliminated through mass drug administration (MDA), or other interventions.

**Table 2:** Global Burden of Major Neglected Tropical Diseases as Estimates of Disability-Adjusted Life Years (DALYs), Years of Life Lost (YLL), and Years Lost Due to Disability (YLD) in WHO Member States.

Disease	2015 Data ^a^			2010 Data ^b^
	YLL (thousand)	YLD (thousand)	DALY = YLL + YDL (thousand)	DALY (thousand) ^b^
Soil-transmitted helminthiasis	449.50	3993.97	4443.47	5043
Ascariasis	225.30	869.37	1094.67	1254
Trichuriasis ^c^	-	542.80	542.80	630
Hookworm ^c^	-	1739.58	1739.58	3159
Schistosomiasis	1042.20	2471.65	3513.85	3971
Dengue fever	1848.79	761.29	2610.08	1243
Lymphatic filariasis ^c^	-	2070.85	2070.85	2740
Cysticercosis	1258.27	598.09	1856.36	503
Rabies	1672.03	0.14	1672.17	2297
Leishmaniasis	1310.74	45.72	1356.46	3754
Onchocerciasis ^c^	-	1135.57	1135.57	564
Foodborne trematodiasis	224.12	842.22	1066.34	665
Echinococcosis	568.20	73.23	641.43	600
Leprosy	457.67	30.97	488.64	215
Human African trypanosomiasis	368.68	2.97	371.65	1346
Trachoma ^c^	-	278.97	278.97	308
Chagas disease	189.65	63.05	252.70	499

^a^ Data source: WHO Health Statistics and Information Systems, 2015 [75]. Data on DALY are not available for Buruli ulcer disease; ^b^ Bhutta et al. (2014) [76]; ^c^ No data available for YLL.
